# Hemolytic uremic syndrome as the presenting manifestation of *WT1* mutation and Denys-Drash syndrome: a case report

**DOI:** 10.1186/s12882-017-0643-1

**Published:** 2017-07-18

**Authors:** Joseph L. Alge, Scott E. Wenderfer, John Hicks, Mir Reza Bekheirnia, Deborah A. Schady, Jamey S. Kain, Michael C. Braun

**Affiliations:** 1 Baylor College of Medicine, Department of Pediatrics, Pediatrician-Scientist Training and Development Program, Houston, TX USA; 2 Baylor College of Medicine, Department of Pediatrics, Renal Section, Houston, TX USA; 3 Baylor College of Medicine, Department of Pathology and Immunology, Houston, TX USA; 4 Baylor College of Medicine, Department of Molecular and Human Genetics, Houston, 77030 USA; 5 Machaon Diagnostics, Inc., Oakland, CA USA; 6 Texas Children’s Hospital, Houston, TX USA

**Keywords:** Hemolytic uremic syndrome, HUS, Wilms tumor 1, WT1, Denys-Drash syndrome, Diffuse mesangial sclerosis

## Abstract

**Background:**

Hemolytic uremic syndrome (HUS) can occur as a primary process due to mutations in complement genes or secondary to another underlying disease. HUS sometimes occurs in the setting of glomerular diseases, and it has been described in association with Denys-Drash syndrome (DDS), which is characterized by the triad of abnormal genitourinary development; a pathognomonic glomerulopathy, diffuse mesangial sclerosis; and the development of Wilms tumor.

**Case presentation:**

We report the case of a 46, XX female infant who presented with HUS and biopsy-proven thrombotic microangiopathy. Next generation sequencing of genes with known mutations causative of atypical HUS found that she was homozygous for the *Complement Factor H* H3 haplotype and heterozygous for a variant of unknown significance in the *DGKE* gene. Whole exome sequencing identified a *de novo* heterozygous *WT1* c.1384C > T; p.R394W mutation, which is classically associated with Denys-Drash syndrome (DDS). At the time of bilateral nephrectomy five months after her initial biopsy, she had diffuse mesangial sclerosis, typical of Denys-Drash syndrome, without evidence of thrombotic microangiopathy.

**Conclusion:**

This unique case highlights HUS as a rare but important manifestation of *WT1* mutation and provides new insight into the genetics underlying this association.

## Background

Hemolytic uremic syndrome (HUS) consists of the triad of hemolytic anemia, thrombocytopenia, and acute kidney injury (AKI). Pathogenic mutations in complement-alternative pathway genes and complement factor H autoantibodies are the most common causes of primary HUS (atypical HUS). [1] However, our limited understanding of the pathogenesis of atypical HUS is highlighted by the relatively high percentage (at least 33%) of cases of atypical HUS with no identifiable underlying cause. [2, 3] The recent discovery of pathogenic recessive mutations in the *DGKE* gene in patients diagnosed with atypical HUS underscores the interaction between complement dysregulation and mutations in non-complement genes (secondary HUS). [4, 5] HUS often occurs secondary to another underlying disease process, classically following a diarrheal illness due to a Shiga toxin-producing bacterial infection. However, it has been associated with a number of other conditions, and a case series by Manenti et al. highlighted the association between HUS and underlying glomerulopathies. [6] We report here the case of a 46, XX female infant who presented with HUS and biopsy-proven thrombotic microangiopathy (TMA) in the setting of a heterozygous mutation in the *Wilms tumor 1 (WT1)* gene that is the most common cause of Denys-Drash syndrome (DDS), which includes a pathognomonic glomerulopathy, diffuse mesangial sclerosis. [7, 8] The purpose of this case report is to highlight the association between HUS and the glomerulopathy caused by *WT1* mutations observed in DDS.

## Case presentation

A previously healthy 8-month-old female presented with three days of vomiting without diarrhea or an antecedent illness. At presentation she was severely hypertensive, volume overloaded, and oliguric. Laboratory workup revealed the triad of hemolytic anemia, thrombocytopenia, and AKI, consistent with TMA. Urinalysis was significant for 3+ hematuria and 4+ proteinuria. Serum chemistry was notable for hyponatremia, hyperkalemia, hyperphosphatemia, and metabolic acidosis. Serum complement C3 was decreased (52 mg/dL; normal value: 86–179 mg/dL), but C4 was normal (17 mg/dL; normal value: 14–45 mg/dL). Platelet activation studies demonstrated increased ATP secretion in response to adenosine diphosphate and arachidonic acid, but were otherwise normal. ADAMST13 activity was 59%, and therefore the diagnosis of atypical HUS was made.

**Fig. 1:**
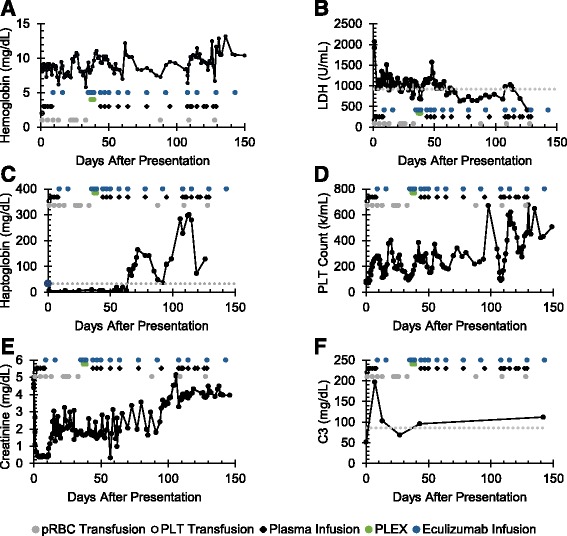
Trend of labs used to monitor for response to treatment. This figure depicts the patient’s therapeutic response to various interventions, including eculizumab infusions, plasma infusions, and plasma exchange, and transfusion of blood products. The patient was routinely monitored for evidence of (**a**-**c**) hemolysis, (**d**) thrombocytopenia and (**f**) complement activation. The upper limit of normal LDH is shown by the dashed line, as is the lower limit of normal for haptoglobin and C3. As shown by her ongoing anemia and thrombocytopenia, the need for numerous pRBC transfusions, and her persistently low haptoglobin and elevated lactate dehydrogenase, the patient did not initially respond to eculizmab therapy. Her clinical status improved following three days of plasma exchange followed by more aggressive twice weekly plasma + eculizumab infusions. However, her (**e**) renal function never recovered and she required chronic renal replacement therapy. LDH, lactate dehydrogenase; pRBC, packed red blood cells; PLEX, plasma exchange; PLT, platelet

**Fig. 2:**
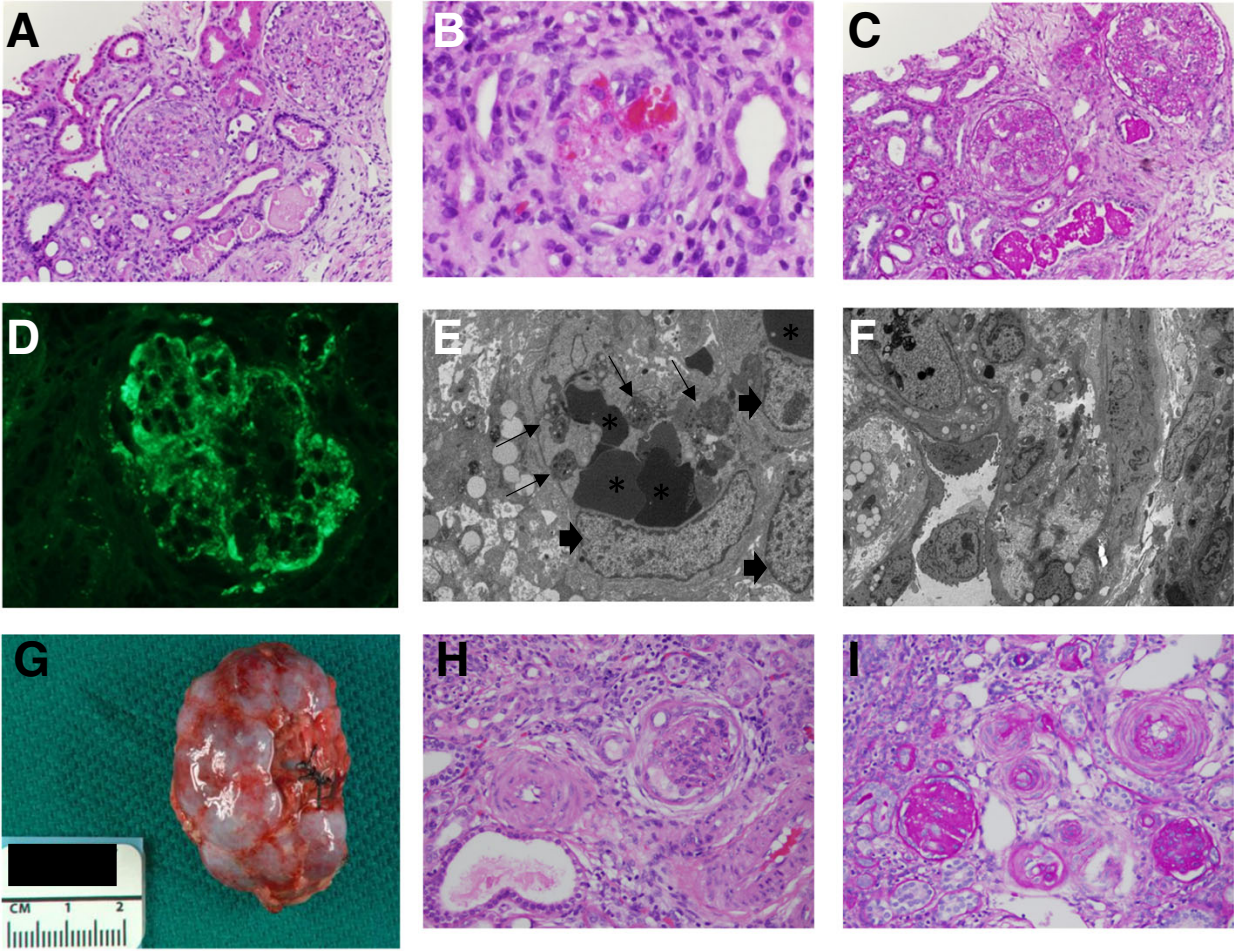
Histopathology of (**a-f**) renal biopsy and (**g-i**) nephrectomy specimens. H&E staining showed **a** mild glomerular hypercellularity and **b** platelet thrombi within capillary loops and the mesangium. **c** PAS staining demonstrated increased matrix production glomeruli without sclerosis. **d** Immunofluorescent antibody staining showed fibrinogen staining within glomerular capillaries. Electron micrographs (EM) demonstrated **e** a thrombus including several platelets (*small arrows*) and erythrocytes (*asterisks*) within a glomerular capillary (endothelial cell nuclei labeled with *large arrows*) **f** a glomerular capillary with adjacent podocyte foot process effacement. Six months after initial presentation the patient underwent bilateral nephrectomy due to the risk of Wilms tumor development. **g** Gross examination of the patient’s kidneys at the time of nephrectomy revealed small, sclerotic kidneys, which had a combined weight of only 30.6 g (average weight for age is 71 g). **h** H&E staining demonstrated a glomerulus undergoing mesangial sclerosis and an adjacent arteriole with “onion skinning” appearance due to intimal and mural thickening. **i**. PAS staining showed globally sclerotic glomeruli and arteriolar lumenal narrowing with intimal and mural thickening

Management included renal replacement therapy, plasma infusions/exchange, and eculizumab infusions. Figure 1 shows the timing of infusions and eculizumab dosing relative to lab trends used to monitor disease activity. During the first week of admission the patient was treated with continuous renal replacement therapy and received daily plasma infusions. On hospital day 8, she was transitioned to intermittent hemodialysis and began a regimen of weekly eculizumab infusions, with all doses administered during the last hour of dialysis, as previously described. [9] After the third eculizumab dose, renal biopsy was performed. Three of eight glomeruli present in the specimen were globally sclerotic. Non-globally sclerotic glomeruli showed mild mesangial cellularity with increased matrix and numerous platelet thrombi visualized within glomerular capillary loops, consistent with active TMA (Fig. 2a-e). Electron microscopy demonstrated podocyte foot process effacement (Fig. 2f). Due to continued evidence of hemolysis (anemia and thrombocytopenia in the setting of low haptoglobin and elevated LDH; Fig. 1a-d) and biopsy-proven active TMA, the patient underwent daily plasma exchange and post-exchange eculizumab redosing for three consecutive days, after which she was started on a regimen of twice weekly intradialytic plasma and eculizumab infusions. Six days after the initiation of combined therapy, hemolysis began to resolve, and complement C3 levels increased to the normal range (Fig. 1a-c, f). Therefore, the frequency of eculizumab dosing was reduced to every 14 days. However, the patient did not recover renal function, and she was eventually transitioned to chronic peritoneal dialysis (Fig. 1e).

**Fig. 3:**
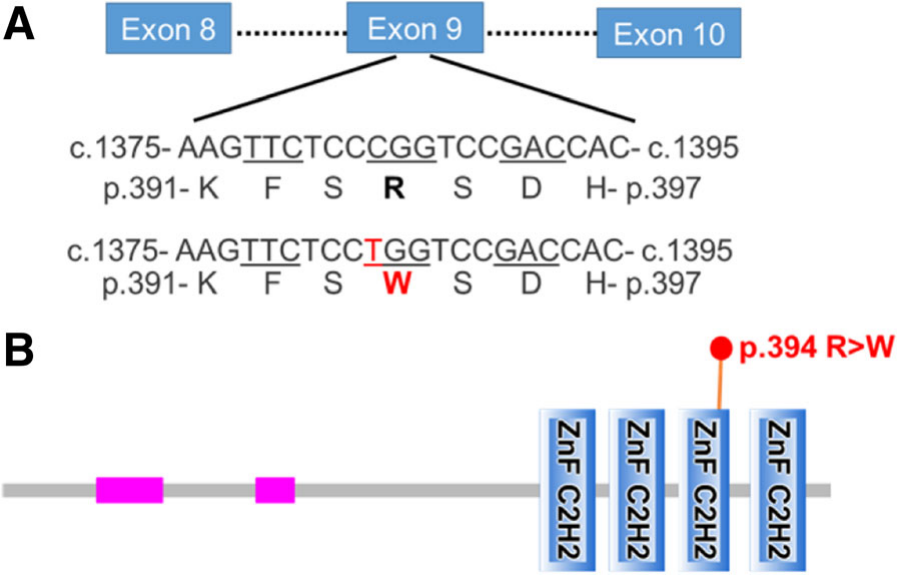
Schematic depicting the location of the patient’s *WT1* mutation. **a** The *WT1* gene encodes a 10 exon mRNA transcript (Transcript ID: ENST00000448076). The patient harbored a c.1384C > T missense mutation in exon 9. **b** The result of this missense mutation is that the corresponding 449 amino acid WT1 protein (Uniprot ID: P19544) has a p.394R > W substitution that is located in the third Zinc finger domain. Pink rectangles represent low complexity regions. Protein schematic was generated using SMART [25]

We performed extensive diagnostic testing to identify the cause of our patient’s HUS. Workup for complement factor H autoantibodies was negative. Neutrophil and lymphocyte expression of membrane cofactor protein, the second most frequently mutated gene associated with atypical HUS, was at the lower limit of normal. [2, 3] We used a commercially available next generation sequencing assay (Machaon Diagnostics, Inc.) to test for mutations in the following twelve genes with known pathogenic mutations associated with atypical HUS: *CFH, MCP, CFI, CFB, C3, CFHR1, CFHR3, CFHR4, CFHR5, thrombomodulin, plasminogen,* and *DGKE. DGKE* sequencing included all exons plus 25 bp of flanking intronic DNA, as well as the previously reported c.888 + 40A > G intronic mutation. [5] The patient was homozygous for the H3 haplotype, which comprises three non-pathogenic SNPs in the *CFH* gene that are associated with predisposition to atypical HUS: (i) c.1–332 C > T in the promoter; (ii) a silent mutation at Q672, and (iii) a E936D substitution. [2, 10] Additionally, our patient was heterozygous for a rare missense SNP in *DGKE* (c.1679A > G; p.Q560R) in the c-terminal low complexity region, which was not thought to be pathogenic since all reported cases of atypical HUS associated with *DGKE* mutations follow a recessive inheritance pattern. [4, 5] Finally, we performed whole exome sequencing using blood samples from the patient and her parents (Baylor Miraca Genetics Laboratories), which identified a *de novo* heterozygous c.1384C > T missense mutation in exon 9 of the *WT1* gene, which was subsequently confirmed by Sanger sequencing. This mutation results in a p.R394W substitution in the third Zinc finger of WT1 protein (Fig. 3), and it is the classic mutation associated with DDS. [7, 8].

DDS is characterized by abnormal genitourinary development in males, Wilms tumor, and diffuse mesangial sclerosis. [11] Karyotyping confirmed that the patient was 46, XX. However, no ovaries were visualized on pelvic ultrasound. Due to the patient’s high risk of developing Wilms tumor, she underwent bilateral nephrectomy five months after initial presentation. Gross examination revealed small, sclerotic kidneys that weighed a combined 30.6 g (average combined weight for age is 71 g; Fig. 2g). Histologic lesions ranged from moderate to severe mesangial hypercellularity with mesangial sclerosis to global glomerulosclerosis (Fig. 2h and i). Arterioles had an “onion skin” appearance with intimal and mural thickening (Fig. 2h and i). There was no evidence of lesions associated with TMA. The histopathologic features were entirely consistent with the diagnosis of diffuse mesangial sclerosis, the glomerulopathy classically associated with DDS. As there was no evidence of diffuse mesangial sclerosis on the initial renal biopsy, these findings show that HUS manifested prior to the development of glomerulopathy in this patient.

## Discussion and Conclusions

We report here the case of a 46, XX infant female who presented with clinical and biopsy features of severe HUS and was subsequently found to harbor a *de novo* heterozygous *WT1* c.1384C > T; p.R394W missense mutation. WT1 is an important transcriptional and post-transcriptional regulator of gene expression and serves as a master regulator of the mesenchymal-to-epithelial transition that is essential for nephrogenesis, although its expression in the developed kidney is limited to podocytes. [12] The *WT1* gene encodes a protein with four C-terminal Krüppel-type Zinc finger domains, which has 36 different isoforms. [13] WT1 binds DNA response elements and functions as a transcriptional activator or repressor through association with epigenetic modifiers. [12] Additionally, WT1, predominantly KTS (+) isoforms, can bind mRNA transcripts and is thought to regulate splicing. [14, 15] Of relevance to this case, the WT1 p.R394W mutation, which is the most common mutation found in patients with DDS, occurs in the third Zinc finger and has been shown to alter the WT1 DNA binding capacity and increase WT1 co-localization with splicing factors, leading to perturbed expression of several genes implicated in glomerulosclerosis. [7, 8, 14, 16, 17].

Our patient’s *WT1* mutation is the most common cause of DDS, a diagnosis that is supported by the findings of diffuse mesangial sclerosis at the time of nephrectomy. [8] Therefore, this case report is further evidence of an apparent association between HUS and DDS that dates back to the original case described by Denys et al., in which features of TMA were noted on histologic analysis of the patient’s nephrectomy specimen. [18] Three cases of HUS associated with DDS in 46, XY males have been previously reported. [19, 20] Manivel et al. reported the case of a 26-month-old 46, XY male with ambiguous genitalia who presented with HUS. Renal biopsy demonstrated features consistent with membranoproliferative glomerulonephritis that was attributed to chronic HUS. This patient underwent bilateral nephrectomy and renal transplantation at 32 months of age, at which time histologic analysis of his native kidneys demonstrated “diffuse global sclerosis”. Sherbotie et al. reported the cases of two boys who developed HUS in the context of DDS that ultimately resulted in end-stage renal disease requiring dialysis and renal transplantation. The first was a 13-month-old 46, XY male who presented with a *Citrobacter* and *Enterococcus* urinary tract infection and nephrotic syndrome, which progressed to HUS within one month. The second case was a 16-month-old 46, XY male who presented with atypical HUS. Mutations in exon 9 of the *WT1* gene were identified in both patients, and of note, although neither patient had been diagnosed with DDS prior to presentation with HUS, both patients had evidence of abnormal genitourinary development (hypospadias, cryptorchidism, and in one case bifid scrotum). Also of note, both patients had normal C3 levels at presentation. Like the case presented by Manivel, our patient had a renal biopsy with evidence of TMA and a later nephrectomy that demonstrated diffuse mesangial sclerosis, clearly demonstrating that HUS preceded the development of glomerulopathy. As in the cases reported by Sherbotie et al., our patient harbored a mutation in exon 9 of the *WT1* gene. In contrast to prior reports, our patient was a 46, XX female with normal external genitalia, and therefore HUS was the only clinical evidence of her disease at the time of presentation. Another factor that distinguishes our case from others is that our patient was hypocomplementemic at presentation, suggesting dysregulation of the complement alternative pathway.

Our case report benefits from tremendous advances that have implicated mutations in complement genes and podocytopathy in the pathogenesis of HUS, which provide a new lens through which to view the biology of the association between HUS and *WT1* mutation. Unlike previous reports of HUS in the context of DDS, we were able to interrogate our patient’s genome for mutations in complement alternative pathway genes implicated in the pathogenesis of atypical HUS. We cannot exclude the possibility that our patient’s presentation may have been due to the combination of *WT1* mutation and predisposition to HUS conferred by her homozygosity for the *CFH* H3 haplotype. Therefore, this case also adds to several reports of HUS occurring in the context of mutations in complement alternative pathway genes and other pathogenic triggers, including *E. coli* 0157:H7 infection, *Streptococcus pneumoniae* infection, hematopoietic stem cell transplantation, cobalamin C deficiency, and cisplatin toxicity. [21] Additionally, her borderline low levels of MCP and abnormal platelet functional studies suggest a potential susceptibility to complement-mediated injury and a prothrombotic phenotype. It is unclear if her *WT1* mutation could have contributed to these abnormal findings. Regarding podocytopathy, the findings of proteinuria at presentation and podocyte foot process effacement on renal biopsy indicate underlying podocyte injury. This case adds to a growing body of literature linking podocyte dysfunction, glomerulopathy, and HUS. [6, 22] Noris et al. have suggested that loss of podocyte-derived VEGF promotes glomerular endothelial dysfunction, leading to the development of HUS. [22] This is an intriguing hypothesis explaining the mechanism that underlies the relationship between DDS and HUS, as in vitro studies have shown that WT1 regulates *VEGF* expression. [23] This function of WT1 is abrogated by the p.R394W mutation, and studies using kidney tissue samples derived from DDS patients have reported loss of intraglomerular expression of inhibitor-type VEGF 165b. [23, 24] Therefore, we hypothesize that our patient’s *WT1* mutation resulted in a podocytopathy that led to endothelial dysfunction, perhaps due to aberrant expression of VEGF165b and/or other factors, which in combination with her homozygosity for the *CFH* H3 haplotype culminated in the development of HUS.

## Abbreviations

AKI: Acute kidney injury
DDS: Denys-Drash syndrome
HUS: Hemolytic uremic syndrome
TMA: Thrombotic microangiopathy
WT1: Wilms tumor 1
